# ‘Whole-Body’ Perspectives of Schizophrenia and Related Psychotic Illness: miRNA-143 as an Exemplary Molecule Implicated across Multi-System Dysfunctions

**DOI:** 10.3390/biom14091185

**Published:** 2024-09-20

**Authors:** John L. Waddington, Xiaoyu Wang, Xuechu Zhen

**Affiliations:** 1 School of Pharmacy and Biomolecular Sciences, RCSI University of Medicine and Health Sciences, D02 YN77 Dublin, Ireland; 2 Jiangsu Key Laboratory of Translational Research and Therapy for Neuro-Psychiatric-Disorders, Department of Pharmacology, College of Pharmaceutical Sciences, Soochow University, Suzhou 215123, China; cpuwxy@126.com (X.W.); zhenxuechu@suda.edu.cn (X.Z.)

**Keywords:** schizophrenia, psychotic illness, whole-body illness, miRNA-143, pathobiology, cardiovascular disease, metabolic syndrome and diabetes, immunity and inflammation, cancer, gut–brain axis

## Abstract

A wide array of biological abnormalities in psychotic illness appear to reflect non-cerebral involvement. This review first outlines the evidence for such a whole-body concept of schizophrenia pathobiology, focusing particularly on cardiovascular disease, metabolic syndrome and diabetes, immunity and inflammation, cancer, and the gut–brain axis. It then considers the roles of miRNAs in general and of miRNA-143 in particular as they relate to the epidemiology, pathobiology, and treatment of schizophrenia. This is followed by notable evidence that miRNA-143 is also implicated in each of these domains of cardiovascular disease, metabolic syndrome and diabetes, immunity and inflammation, cancer, and the gut–brain axis. Thus, miRNA-143 is an exemplar of what may be a class of molecules that play a role across the multiple domains of bodily dysfunction that appear to characterize a whole-body perspective of illness in schizophrenia. Importantly, the existence of such an exemplary molecule across these multiple domains implies a coordinated rather than stochastic basis. One candidate process would be a pleiotropic effect of genetic risk for schizophrenia across the whole body.

## 1. Introduction

Over the past half century, research on schizophrenia has generated a continually evolving landscape, with contemporary reviews documenting both concepts and controversies in relation to phenomenology, taxonomy, epidemiology [1], genetics [2], brain pathobiology [3], and treatment [4,5,6]. Among evolving themes, there is a shift from a schizophrenia-centric to broader transdiagnostic, dimensional-spectrum perspectives of psychotic illness [7,8,9,10] and early intervention to limit progression and, potentially, achieve prevention [7,11,12]. From this point on, ‘schizophrenia’ will be used as a convenient shorthand for such contemporary transdiagnostic, dimensional-spectrum perspectives of psychotic illness.

In parallel, there is the progressive elaboration of a lifetime trajectory model, in which the above domains of impairment are rooted in disturbances of early brain development that can then be sculpted by later phases of development, maturity, and aging [13,14,15,16,17,18,19,20]. However, two recent articles [21,22] have reviewed evidence suggesting that schizophrenia is associated with a broader range of biological abnormalities that extend beyond the brain across various bodily regions and functionalities to include what would otherwise be thought of as general medical conditions. Furthermore, these abnormalities are found in regions and functionalities that may be related to the marked reduction in life expectancy in schizophrenia [23,24,25,26]. Three further articles [27,28,29] relate to this theme of mal-development in its broadest sense by reviewing evidence for schizophrenia being a disorder of accelerated aging that extends beyond the brain on a whole-system basis.

While the present article outlines the evidence for a whole-body perspective of schizophrenia, there is no credible evidence for any unitary and ubiquitous ‘cause’ of this disorder. For example, to date, 287 genomic loci have been reported to show variant associations with risk for schizophrenia, among which 106 are protein-coding, with enrichment for genes implicated by such variants in other neurodevelopmental disorders [2]. Furthermore, there is no pathognomonic feature at the level of either psychopathology or pathobiology [1]. Therefore, any whole-body perspective must have alternative explanations, which may include (independently or in combination) the following:

(i) The diversity of genomic loci reported to show variant associations with risk for schizophrenia, which may include loci also associated with risk for dysfunction external to the brain; this may involve pleiotropic effects via one or more regulatory molecules distributed across other bodily regions and dysfunctions;
(ii) Body systems that during early fetal life share an intimate embryonic relationship with the nervous system, including one or more regulatory molecules; while these systems subsequently differentiate into the brain and other anatomically and functionally distinct organs, each may retain the shared involvement of one or more of those regulatory molecules and, hence, of dysfunctions associated with their dysregulation. These effects may be modulated by endogenous processes and external events across development from infancy to adolescence–young adulthood and the emergence of the diagnostic symptoms of psychotic illness.

This article first summarizes the evidence for a still controversial whole-body perspective of schizophrenia across several systems external to the brain; more detailed findings relating to each system can be found in two recent reviews [21,22]. Second, it introduces the regulatory molecule miRNA-143 and reviews emerging evidence for its role in brain processes fundamental to the epidemiology and pathobiology of schizophrenia. Third, it reviews emerging evidence for the involvement of miRNA-143 in dysfunction in each of the several body systems external to the brain and offers conclusions.

## 2. Evidence for a ‘Whole-Body’ Concept of Schizophrenia

When considering abnormalities in schizophrenia that involve regions and systems external to the brain, it is necessary to resolve for each instance at least two following issues: (i) whether this is primarily a peripheral manifestation of a centrally-mediated process, for example, in movement disorder; (ii) whether this is secondary to peripheral effects of treatment with antipsychotic drugs, for example on the cardiovascular system. Antipsychotics are almost invariably administered consequently to clinical presentation with first-episode psychosis (FEP), hence the opportunity to assess untreated illness is typically very brief in the developed world [21,22].

### 2.1. Abnormalities of Surface Anatomy and Cardiovascular Structure and Function

The above criteria (i) and (ii) indicate in schizophrenia an excess of surface anatomical abnormalities, particularly of regions of the body that share an intimate embryological relationship to the brain, that are intrinsic to this disorder. These extend from congenital anomalies to minor physical anomalies, typically of the craniofacial area, particularly of the frontonasal prominences, but extending across the body to the hands and feet [22,30,31]. Under these surface abnormalities, cardiac magnetic resonance imaging has revealed in schizophrenia smaller left and right ventricular end-diastolic volume, end-systolic volume and stroke volume, unaltered ejection fractions, and larger ventricular concentricity and septal thickness; these abnormalities in schizophrenia are evident in the absence of risk factors for cardiovascular disease and after controlling for socioeconomic status, lifestyle, and medication [32]. Notably, such adverse cardiac outcomes are reported to be associated with high polygenic risk scores for schizophrenia [33].

### 2.2. Metabolic Syndrome and Diabetes

Schizophrenia is associated with medical comorbidities that are an important determinant of reduced life expectancy and include an increased risk for metabolic syndrome with an associated increase in risk for heart disease and type 2 diabetes [23,24,25]. These abnormalities include elevated fasting plasma glucose levels, plasma glucose levels after an oral glucose tolerance test, fasting plasma insulin levels, insulin resistance, triglyceride levels, reduced total cholesterol, and low and high-density lipoprotein cholesterol levels [34,35,36,37,38]. Additionally, in schizophrenia, the risk for a family history of type 2 diabetes is double that among the general population [39], suggesting shared genetic risk.

### 2.3. Immune–Inflammatory Processes

Elevations in peripheral inflammatory proteins, particularly blood cytokine levels, are consistently reported in schizophrenia; these include interleukin (IL)-1β, IL-1 receptor antagonist, soluble interleukin-2 receptor, IL-6, IL-8, IL-10, tumor necrosis factor-α, and C-reactive protein [40,41]. Magnetic resonance imaging findings in schizophrenia relating to cardiac concentricity as a predictor of cardiovascular disease show evidence of adipose dysfunction; adiponectin is reduced, together with elevated levels of triglycerides, C-reactive protein, and fasting glucose [42]. These findings integrate inflammatory processes with cardiac anatomy (see Section 2.1) and metabolism (see Section 2.2) within a whole-body concept of abnormality in schizophrenia.

Elevations in peripheral immune cells are also consistently reported in schizophrenia: in blood, these include leukocyte count, granulocyte count, neutrophil granulocyte count, monocyte count, and B lymphocyte count; additionally, neutrophil/lymphocyte, monocyte/lymphocyte, and platelet/lymphocyte ratios are elevated [43]. These findings indicate a broad activation of the immune system in schizophrenia, with cells from both the myeloid and the lymphoid line being elevated.

### 2.4. Malignant Disease

Recent meta-analyses on schizophrenia indicate increased mortality due to breast, colon, and lung cancer [44], increased risk for breast, cervical, colorectal, oesophageal, testicular, and uterine cancer, with increased risk for metastases at diagnosis, and decreased risk for prostate, skin, and thyroid cancer [45]. While these associations may be modulated by both personal lifestyle and health service-related factors, their robustness focuses on the possibility of overlapping genetic risk. Among risk genes for schizophrenia [2], there are associations with increased risk for breast, ovarian, thyroid, lung, and colorectal cancers and decreased risk for stomach cancer [46]. These associations suggest that risk genes for schizophrenia may exert malignant effects across the whole body.

### 2.5. Gut–Brain Axis

Over recent decades, studies have indicated in depth how the gastrointestinal tract (GIT) and microbiota influence central nervous system (CNS) function and how the CNS influences GIT function and microbiota [47,48]. Genetic correlations have been examined across the GIT disorders of inflammatory bowel disease, irritable bowel syndrome, peptic ulcer disease, gastroesophageal reflux disease, and the six psychiatric disorders of schizophrenia, bipolar disorder, major depressive disorder, attention-deficit/hyperactivity disorder, posttraumatic stress disorder, and anorexia nervosa [49]. The findings for schizophrenia include correlations both across and between these GIT and CNS disorders, indicating that risk genes for schizophrenia may exert effects across the whole body. Associations between intestinal inflammation and psychotic symptom severity [50] and between the gut microbiota/microbiome–metabolome, disruption to brain connectivity, and polygenic risk score for schizophrenia [51] elaborate these findings.

## 3. miRNAs and Schizophrenia

Recent decades have seen a rapid expansion in research on microRNAs (miRNAs). This class of small, noncoding RNAs regulates the expression of genes at the post-transcriptional level, and miRNAs have been shown to play a role in regulating numerous fundamental cellular processes, including development, proliferation, differentiation, and apoptosis. It is notable that circulating miRNAs are involved not only in normal biological processes but have also been implicated in the pathobiology of numerous disorders, including cardiovascular diseases, diabetes, cancer, and neuropsychiatric disorders [52,53].

Recently, the role of miRNAs in schizophrenia has attracted attention [54,55,56]. In a recent meta-analysis of miRNAs in schizophrenia [57], 22 were found to be up-regulated, and 5 were found to be down-regulated; furthermore, the application of the deep (computational) learning method Stacked AutoEncoder for potential miRNA-Disease Association prediction (SAEMDA) identified 20 additional candidate miRNAs for association with schizophrenia. The target genes were associated particularly with functions relating to cell signaling, cancers, cell death, endocrine disorders, kinase activities, oxidative stress, prenatal infections, and transcription regulation.

### 3.1. miRNA-143 and Risk for Schizophrenia

The 20 candidate miRNAs identified using SAEMDA included miRNA-143.68, which was not identified as differentially expressed in schizophrenia in the meta-analysis of studies published up to early 2022 [57]. However, a subsequent study has reported functional variants of miR-143 to be associated with schizophrenia susceptibility [58]. More specifically, the link between rs4705342T/C and rs4705343T/C polymorphisms in the promoter region of miR-143 and risk for schizophrenia was investigated in 202 cases and 196 control subjects. The genotypic analysis of rs4705342T/C showed that the CC genotype in the codominant model was associated with a reduced risk for schizophrenia. Additionally, reduced risk for schizophrenia was observed under allelic, dominant, and recessive models of this variant. Regarding rs4705343T/C, an increased risk for schizophrenia was found under the codominant CC and recessive contrasted genetic models. These heuristic findings prompt consideration of the molecular biology of miRNA-143, the breadth and depth of its involvement in schizophrenia, and processes related to schizophrenia.

### 3.2. Molecular Biology of miRNA-143

miR-143 is a highly conserved miRNA located in the region of chromosome 5q32 in the human genome [59,60]. Initially, primary miR-143 is transcribed by RNA polymerase II in the nucleus and then processed by the class 2 ribonuclease III enzyme Drosha into precursor miRNA. Subsequently, precursor miR-143 is exported into the cytoplasm and trimmed by the endoribonuclease Dicer to generate two mature forms: guide strand miR-143-3p and passenger strand miR-143-5p. Either strand can be selected for RNA-induced silencing complex loading, which allows for the seed region at the 5′ end of the miRNA to bind to the 3′-untranslated region of target mRNA transcripts, thereby suppressing protein expression by post-transcriptional regulation [52,60]. As miR-143-3p and miR-143-5p do not share the same seed region, this may imply that they have different functional roles. Interestingly, in studies on miRNA-143 in relation to schizophrenia (see below), both experimental findings in animal models and clinical findings in schizophrenia patients more commonly implicate miR-143-3p, with only limited information concerning a role for miR-143-5p.

### 3.3. miRNA-143 and Pathobiological Mechanisms Related to Schizophrenia

We have recently reported findings using the administration of the psychotomimetic agent phencyclidine (PCP), a non-selective antagonist of the N-methyl-D-aspartate (NMDA) glutamate receptor [61]. In this widely studied animal model of schizophrenia-related processes, deriving from the glutamate hypofunction hypothesis of schizophrenia, next-generation microRNA deep-sequencing revealed acute PCP administration in mice to alter the expression profiles of miRNAs from mouse astrocytes in the prefrontal cortex, with the most robust reduction seen for miR-143; this effect was also seen in plasma. The reduced miR-143 expression following acute administration of PCP was evident in the prefrontal cortex and hippocampus but not in the striatum; these effects were antagonized by the antipsychotics haloperidol and clozapine and by the selective dopamine D2 receptor antagonist eticlopride but not by the selective dopamine D1 receptor antagonist SCH23390. A subsequent study involving repeated injection of MK-801, another non-selective antagonist of the NMDA receptor, has confirmed our original report of astrocytic downregulation of hippocampal miRNA-143 [62]. 

Furthermore, in HT-22 cells and primary cortical cultured neurons, we found that stimulation of D2 dopamine receptors with the selective D2 agonist quinpirole decreased expression of miRNA-143, and this effect was antagonized by eticlopride, indicating that the D2 receptor directly regulated the expression of miRNA-143 [61]. It is notable that increased presynaptic dopamine synthesis is the most reproducible clinical pathobiological finding in schizophrenia and that antagonism of D2 receptors is the primary action of all first- and second-generation antipsychotic drugs [3].

Additionally, we demonstrated that miRNA-143 directly targeted the 3′ untranslated region of neuregulin-1 (NRG1) mRNA to reduce the protein expression of NRG1 in HT-22 cells and that administration of the D2 receptor agonist quinpirole to mice enhanced expression of NRG1 in the prefrontal cortex [61]. NRG1 is a developmental regulator that has long been implicated in the pathobiology of schizophrenia [63,64,65].

### 3.4. miRNA-143 and Clinical Response to Antipsychotics in Schizophrenia

Unbiased RNA sequencing analysis has been performed using homogeneous populations of neural stem cells derived from induced pluripotent stem cells of three patients with schizophrenia who responded to the antipsychotic olanzapine and four patients who did not respond to olanzapine [66]. Lack of responsivity to olanzapine was associated with NRG-1 deficiency-induced mitochondrial dysfunction. Furthermore, NRG-1 knockout mice showed schizophrenia-related behavioral deficits that were less responsive to amelioration by olanzapine than similar behavioral deficits induced by MK801. Notably, miRNA-143-3p was a critical NRG-1 target related to mitochondrial dysfunction. More specifically, miR143-3p levels in neural stem cells were associated with a lack of clinical responsivity to olanzapine. Additionally, olanzapine resistance in NRG-1 knockout mice could be rescued by treatment with miRNA-143-3p agomir. These observations elaborate our original findings [61] by additionally providing direct evidence that olanzapine resistance is associated with NRG-1 deficiency-induced mitochondrial dysfunction. In so doing, they link the effects of olanzapine and NRG-1 deficiency-induced mitochondrial dysfunction to an NRG-1/miRNA-143-3p nexus that is associated with developmental pathobiology in schizophrenia and clinical response to antipsychotic drugs.

### 3.5. miRNA-143 in Disorders Related Phenomenologically to Schizophrenia

While psychotic-like experiences are well-documented to occur among the general population without a psychotic illness, their persistence is a risk factor for psychiatric diagnoses, including schizophrenia [67]. Recently, subjects with psychotic-like experiences were found to show downregulation of miRNA-143-3p [68].

It is well established that psychosis is more common in subjects with temporal lobe epilepsy than among the general population and can appear very similar to the psychosis of schizophrenia prior to neuropsychiatric evaluation [69]. Subjects with temporal lobe epilepsy have been found to show dysregulation of miRNA-143 [70].

## 4. miRNA-143 and Whole-Body Aspects of Schizophrenia

### 4.1. miRNA-143 and Cardiovascular Disease

Similar to abnormalities of cardiovascular structure and function in schizophrenia (see Section 2.1), studies indicate that miRNA-143 is dysregulated in domains of cardiovascular disease that overlap with those evident in schizophrenia [71,72]. More specifically, miRNA-143 is dysregulated in left ventricular dysfunction [73], an abnormality noted also in schizophrenia [32].

### 4.2. miRNA-143, Metabolic Syndrome, and Diabetes

Similar to schizophrenia being associated with an increased risk for metabolic syndrome with an associated increase in risk for heart disease and diabetes (see Section 2.2), studies indicate miRNA-143 to be dysregulated in metabolic syndrome [74,75] and diabetes [76,77]. The role of miRNA-143 in these processes includes targeting the insulin-like growth factor 2 receptor [78].

### 4.3. miRNA-143, Immunity, and Inflammation

Similar to abnormalities of immune–inflammatory processes being found in schizophrenia (see Section 2.3), studies indicate miRNA-143 to be dysregulated in immune–inflammatory processes [79]. For example, schizophrenia is associated with an increased risk for inflammatory bowel disease [80], in which miRNA-143 is known to be dysregulated [81] and may target autophagy-related 2B to influence autophagy and inflammatory responses [82].

### 4.4. miRNA-143 and Malignant Disease

Similar to schizophrenia being associated with increased risk for breast and colorectal cancer (see Section 2.4), studies indicate that miRNA-143 is dysregulated in breast cancer [59,83,84] and colorectal cancer [85,86]. miRNA-143 may be more involved in the development than in the progression of cancer through influences on apoptosis and the integrity of the cell cycle [87].

### 4.5. miRNA-143 and the Gut–Brain Axis

Similar to how the gut–brain axis is implicated in schizophrenia (see Section 2.5), experimental studies implicate miRNA-143 in the course of gut microbiota colonization of the hypothalamus in porcine neonates [88]. This indicates the involvement of miRNA-143 expression in a developmental process whereby gut microbiota influence brain function. 

## 5. Conclusions

Of course, miRNAs other than miRNA-143 are implicated in processes related to schizophrenia, and miRNA-143 is implicated in general medical and psychiatric disorders other than schizophrenia [52,53,54,55,56,57]. Nevertheless, as summarized in Table 1, across this continually expanding research front, it is striking that miRNA-143 is implicated not only in processes fundamental to the epidemiology and pathobiology of schizophrenia; it is also implicated in each of the main domains of abnormality that are external to the brain and constitute the body of evidence for a still controversial whole-body perspective of illness in schizophrenia [21,22]. These findings relating to miRNA-143 and deriving from studies of varying sample sizes (extending from limited to large) collectively imply a coordinated rather than stochastic process and raise the likelihood of a broader range of molecules being involved. One candidate process would be a pleiotropic effect of genetic risk for schizophrenia across the whole body. 

While the mechanisms and pathways by which miRNA dysregulation might influence processes underlying schizophrenia continue to evolve [52,53,57,89,90], this is occurring on a broader landscape. It appears that polygenic risk scores for schizophrenia are also associated with poorer overall health, more hospital inpatient diagnoses, and more individual illnesses that extend beyond mental illness to yet broader domains of bodily dysfunction [91]. These considerations are related to the still evolving concept of a general disease (‘d’) factor underlying the comorbidity of mental and physical illness as an elaboration of a general psychopathology factor (‘p’) underlying mental illness [92]. Within this framework, miRNA-143 could be interpreted as exemplifying a molecule associated with such a putative ‘d’ factor.

## Figures and Tables

**Table 1:** Domains of ‘whole-body’ abnormality in schizophrenia in relation to miRNA-143 findings.

Domain of Abnormality in Schizophrenia	miRNA-143 Findings
Risk for schizophrenia	miRNA-143 implicated in clinical risk for schizophrenia
Brain pathobiology	miRNA-143 implicated in the most widely adopted rodent model of schizophrenia
Therapeutic response to antipsychotics	miRNA-143 implicated in the most widely adopted rodent model of schizophrenia and therapeutic response in schizophrenia
Cardiovascular disease	miRNA-143 implicated in cardiovascular disease
Metabolic syndrome and diabetes	miRNA-143 implicated in metabolic syndrome and diabetes
Immune–inflammatory disorder	miRNA-143 implicated in immune–inflammatory disorder
Malignant disease	miRNA-143 implicated in malignant disease
Gut–brain axis dysfunction	miRNA-143 implicated in porcine model of gut–brain axis dysfunction
